# Characterizations of alveolar repair after mandibular second molar extraction: an experimental study in rats

**DOI:** 10.1590/1678-7757-2022-0010

**Published:** 2022-07-08

**Authors:** Jianbin Li, Zhenxian Sheng, Jing Sun, Ronglin Wang, Xijiao Yu

**Affiliations:** 1 Binzhou Medical College School of Stomatology Shandong China Binzhou Medical College, School of Stomatology, Shandong, China.; 2 Central Laboratory of Jinan Stomatological Hospital Jinan Key Laboratory of Oral Tissue Regeneration Department of Endodontics Shandong Province China Central Laboratory of Jinan Stomatological Hospital, Jinan Key Laboratory of Oral Tissue Regeneration, Department of Endodontics, Shandong Province, China.; 3 Central Laboratory of Jinan Stomatological Hospital Jinan Key Laboratory of Oral Tissue Regeneration Department of Periodontology Shandong Province China Central Laboratory of Jinan Stomatological Hospital, Jinan Key Laboratory of Oral Tissue Regeneration, Department of Periodontology, Shandong Province, China.; 4 Central Laboratory of Jinan Stomatological Hospital Jinan Key Laboratory of Oral Tissue Regeneration Department of Prosthodontics Shandong Province China Central Laboratory of Jinan Stomatological Hospital, Jinan Key Laboratory of Oral Tissue Regeneration, Department of Prosthodontics, Shandong Province, China.

**Keywords:** Socket healing, Alveolar bone healing, Tooth extraction, Site preservation

## Abstract

Characterizations of rat mandibular second molar extraction socket with significantly different buccal and lingual alveolar ridge width remain unclear.

**Objective:**

To observe alterations in the alveolar ridge after extraction of mandibular second molars, and to examine processes of alveolar socket healing in an experimental model of alveolar ridge absorption and preservation.

**Methodology:**

Eighteen Wistar rats were included and divided into six groups regarding healing time in the study. Bilateral mandibular second molars were extracted. The rats with tooth extraction sockets took 0, 1.5, 2, 3, 4 and 8 weeks of healing. Histological observation, tartrate-resistant acidic phosphatase (TRAP) staining, Masson’s trichrome staining, immunohistochemical staining and micro-computed tomography (micro-CT) were applied to estimate alterations in the alveolar ridge.

**Results:**

Different buccal and lingual alveolar ridge width led to different height loss. Lingual wall height (LH) decreased significantly two weeks after tooth extraction. Buccal wall height rarely reduced its higher ridge width. From two to eight weeks after extraction, bone volume (BV/TV), density (BMD), and trabecular thickness (Tb.Th) progressively increased in the alveolar socket, which gradually decreased in Tb.Sp and Tb.N. LH showed no significant change during the same period. Osteogenic marker OCN and OPN increased during bone repair from two to eight weeks. The reduced height of the lingual wall of the tooth extraction socket was rarely repaired in the later repair stage. Osteoclast activity led to absorption of the alveolar ridge of the alveolar bone wall within two weeks after operation. We observed positive expression of EMMPRIN and MMP-9 in osteoclasts that participated in the absorption of the spire region.

**Conclusion:**

Extraction of rat mandibular second molars may help the study of alveolar ridge absorption and preservation. The EMMPRIN-MMP-9 pathway may be a candidate for further study on attenuating bone resorption after tooth extraction.

## Introduction

Dental implantation is an advanced and effective clinical method to repair tooth loss, and it has an excellent aesthetic restoration effect and a high survival rate.^1 , 2^ However, alveolar ridge resorption after tooth extraction, especially the loss of height and width, has been the primary challenge of implant restoration.^3^ ,^4^

Tooth extraction models of different teeth in different animals have been reported, including the extraction of rats’ maxillary incisors,^5 , 6^ maxillary first molars,^7 - 9^ mandibular first molars^10 , 11^ and beagle dogs’ molar extraction.^12 , 13^ Researchers have found useful data from the models. Beagle dogs need four to six months of healing after tooth extraction to show alveolar repair. Histological analysis of 1, 2, 4 and 8 weeks of healing reveals marked osteoclastic activity, which causes resorption of the crestal region of the buccal and the lingual bone wall.^12^ The extraction sockets show an area of minor mineralized bone after three months. The borderline between new and pre-existing bone disappears after six months.^14^ Compared to beagle dogs, rats are cheaper and easier to obtain and raise. Eight weeks of healing is usually applied to observe alveolar repair in rats. Bone healing after extraction in the rats can be divided into the following three stages: the blood clot stage, the connective tissue and new bone formation stage, and the ossification stage, all completed within 28 days.^5 , 15^ Similarly, another study on rats has shown that the extraction socket was gradually filled in the following two weeks from the margins inwards. The socket outline is invisible 30 days after tooth extraction.^16^

Rat mandibular incisor is difficult to extract because its root is long and runs through the mandible, which is a suitable experimental model to study ameloblast activity^17^ and tooth eruption^18^ for continuous growth. The width of the alveolar ridge of rat maxillary incisors, maxillary first molars and mandibular first molars, was similar between the buccal and lingual sides.

However, few studies report the crestal region resorption and alveolar repair in tooth extraction model with significantly different buccal and lingual width. The buccal wall of mandibular second molars was more than twice as thick as the lingual wall. Rat mandibular second molar with three roots is similar to human mandibular first molar, which is one of the most easily lost teeth in humans.^19^ Rat mandibular second molar is easier to extract than rat mandibular first molar with four roots.^20^ Rat mandibular incisor is not applied because its root keeps growing. The high bone density of the rat mandible better characterizes the bone to observe alveolar ridge absorption and repair using micro-computed tomography (micro-CT).

In this study, we extracted bilateral mandibular second molars to observe changes in the alveolar bone wall after tooth extraction and to examine the process of alveolar socket healing. This experimental model can help to assess if rat mandibular second molar extraction is a suitable experimental model to study alveolar ridge absorption and preservation.

Previous studies have suggested that extracellular matrix metalloproteinase inducer (EMMPRIN) and its downstream matrix metalloproteinase-9 (MMP-9) are involved in osteoclast activation and periodontal inflammatory bone destruction.^21^ EMMPRIN is essential in the maturation of dental hard tissue and the formation of an eruption pathway^22^ and gets involved in osteoclastogenesis and alveolar bone resorption during orthodontic tooth relapse.^23^ The expression level of EMMPRIN in the alveolar ridge resorption after tooth extraction is unclear.

In this study, the expression levels of EMMPRIN and MMP-9 in the alveolar ridge absorption were detected by immunohistochemical staining to confirm if EMMPRIN and MMP-9 participate in alveolar ridge absorption after tooth extraction.

## Methodology

### Animals

The animals were maintained on a normal hard food diet with water provided *ad libitum* . The animals were housed in cage racks with a 12 h light/12 h dark cycle (light on from 8:00 AM to 8:00 PM) at room temperature of 22–24°C and 45% relative humidity. During the experiments, all operations related to animal welfare and treatment were conducted in accordance with the Chinese Animal Management Regulations. A small dental elevator was used. The rotation and translation of the elevator offer a wedge and lever action, applied on the mandibular second molars to be extracted.

Eighteen six-week-old male Wistar rats (weight 200-210 g) were included and maintained in the study. Bilateral mandibular second molars were extracted. No medication was applied after extraction to avoid side effect. The twelve rats were euthanized by excessive chloral hydrate 0, 2, 4, and 8 weeks after surgery. The mandibles (n=6) with tooth extraction sockets were separated and immediately fixed in 4% paraformaldehyde for 24 hours and preserved in 70% alcohol for micro-CT, then decalcified in 10% ethylenediaminetetraacetic acid (EDTA) and embedded in paraffin. Other six rats were executed after 1.5 and 3 weeks. The mandibles containing the extraction sockets were dissected, decalcified in 10% ethylenediaminetetraacetic acid (EDTA), and embedded in paraffin. The sections were stained with hematoxylin and eosin, TRAP staining, Masson’s trichrome staining, and immunohistochemical staining. The use of experimental animals was approved by the Animal Care and Use Committee of Jinan Stomatological Hospital (no. JNSKQYY-2019-014).

### Micro-CT detection

The mandibles containing the extraction sockets were assessed three dimensionally using a micro-CT system (SkyScan 1176, Belgium) and scanned at 65 kV/380 µA. The 3D regions of interest (ROI) were selected before the volumetric measurements; rat mandibular second molar extraction sockets were chosen as ROIs. The lingual wall height (LH) of rat mandibular second molar extraction sockets was measured. The scan result of micro-CT applied the percentage of the bone area of ROI as the bone loss area measurement parameter. The CT Analyzer software (Bruker) was used to analyze the microstructural parameters such as percentage of the bone area (bone area/tissue area), trabecular separation (Tb.Sp), trabecular thickness (Tb.Th), trabecular number (Tb.N) and bone mineral density (BMD).

### Hematoxylin and eosin staining

Sections were first deparaffinized by xylene, hydrated in gradient ethanol and treated with hematoxylin for three min to stain cell nucleus. After washing for five min with running water, the sections were reacted with eosin for 30 s.

### Masson’s trichrome staining

Masson’s trichrome was performed with the aid of the Masson staining kit (Jiancheng Biological Inc, Nanjing, China).^24^

### TRAP staining

TRAP staining was performed by using a commercially available kit (Joy Tech Bio. Co., Zhejiang, China).^25^

### Immunohistochemical staining

Streptavidin-Peroxidase kit (Zhongshan, Beijing, China) was used according to the instructions. Polyclonal antibody EMMPRIN and MMP-9 (dilution 1:150, Abcam, UK) were applied. Osteocalcin (OCN) and osteopontin (OPN) (dilution 1:100, Bioss, China) were performed to determine the expression of these proteins in the alveolar bone healing. PBS was obtained as negative control.

### Statistical analysis

All data are expressed as the mean ± standard deviation. Student’s *t* tests, one-way analysis of variance, and the Newman–Keuls test were conducted using the GraphPad Prism 5 software. The differences were considered significant at p<0.05.

## Results

### Observation of rat mandibular second molar crestal region

**Figure 1:**
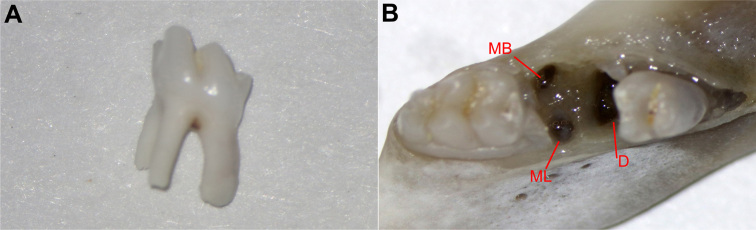
Observation of rat mandibular second molar crestal region. The root morphology of rat mandibular second molars with only three roots was similar to human mandibular first molars. Mandibular second molars were easier to extract compared to the four roots of rat mandibular first molar (A). The crestal region of the lingual bone wall was thinner than that of the buccal bone wall in mandibular second molars (B). MB mesiobuccal, ML mesiolingual, D distal

The root morphology of rat mandibular second molars with only three roots is similar to human mandibular molars ( Figure 1A ). Mandibular second molars were completely extracted. The crestal region of the buccal bone wall is thicker than that of the lingual bone wall in mandibular second molars ( Figure 1B ).

### Observation of rat mandibular second molar extraction sockets by micro-CT

**Figure 2:**
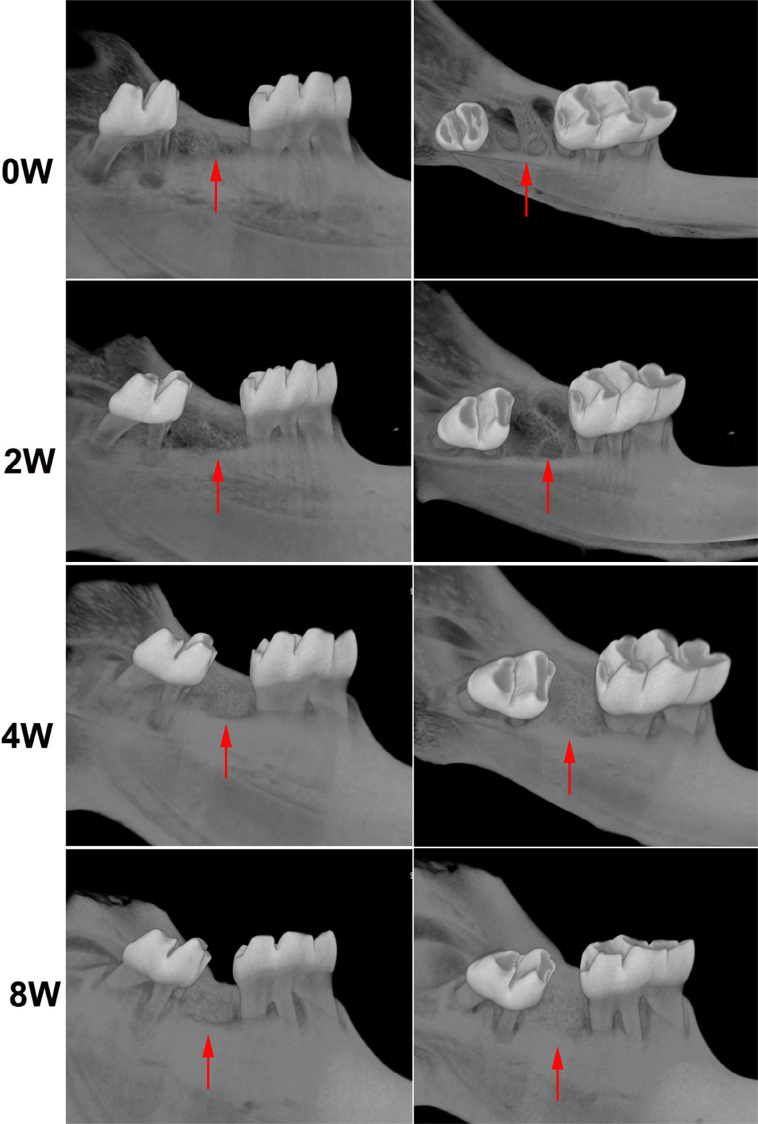
Three-dimensional observation of rat mandibular second molar extraction sockets with 0, 2, 4 and 8 weeks of healing by micro-CT. Three fresh alveolar sockets were observed after tooth extraction. Alveolar socket healing went from the second week to the eighth week. The extraction socket was fully filled with new bone after eight weeks

**Figure 3:**
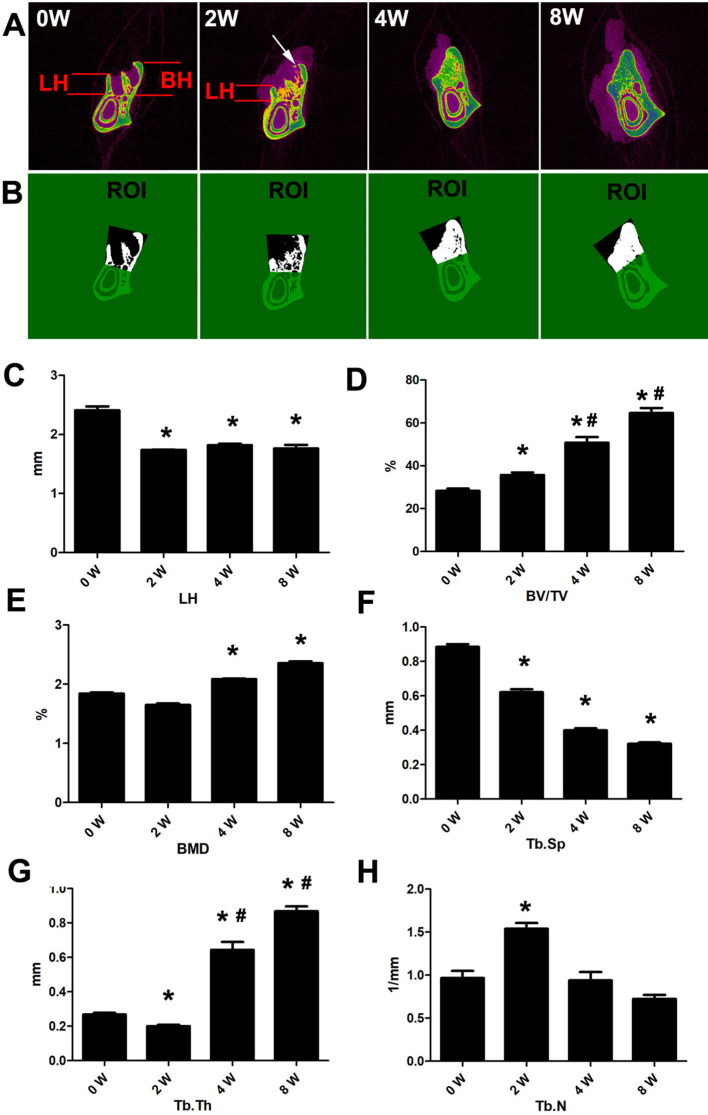
Micro-CT evaluation of alveolar socket of rat mandibular second molar extraction sockets with 0, 2, 4 and 8 weeks of healing. Representative sagittal imaging. Lingual wall height (LH) decreased significantly two weeks after operation. LH showed no significant change from two to eight weeks. Meanwhile, new trabecular bone filling in the apical third of the alveolar socket was detected. Bone resorption in the crestal region of the buccal bone wall was mainly found near the extraction socket side (white arrow). Buccal wall height was rarely reduced after extraction (A and C). Region of interest (ROI) of rat mandibular second molar extraction sockets. Mean values and standard deviation of BV/TV(D), BMD(E), Tb.Th(F), Tb.Sp(G) and Tb.N(H). BV/TV, BMD and Tb.Th increased progressively from two to eight weeks. Consequently, a gradual decrease of Tb.Sp and Tb.N was observed. *Different from 0W (p<0.05), #Different from 2W (p<0.05)

We observed alterations eight weeks after tooth extraction, and three alveolar sockets after rat mandibular second molar extraction. The resorption of the lingual walls of the extraction site occurred after two weeks. The alveolar ridge underwent bone resorption, remodeling and healing. The extraction socket was fully filled with new bone eight weeks after extraction ( Figure 2 ). The lingual wall height (LH) of the extraction sockets decreased significantly two weeks after extraction. Bone absorption in the crestal region of the bone wall near the buccal side was mainly found near the extraction socket side, and buccal wall height was rarely reduced after extraction. Meanwhile, new trabecular bone filling was found in the apical third of the alveolar socket ( Figure 3A - C ).

The alveolar socket had been largely filled with new bone after four weeks. BV/TV, BMD and Tb.Th increased progressively from two to eight weeks after extraction (p<0.05), with a gradual decrease in Tb.Sp and Tb.N (p<0.05). The micro-CT results that showed the bone volume and thickness of trabeculae increased during the alveolar socket healing process (p<0.05) ( Figure 3D - H ). LH showed no significant change from two to eight weeks. The reduced height of the bone wall near the lingual side of the extraction socket was rarely repaired in the later repair stage.

### Expression levels of OCN and OPN during bone repair from two to eight weeks

**Figure 4:**
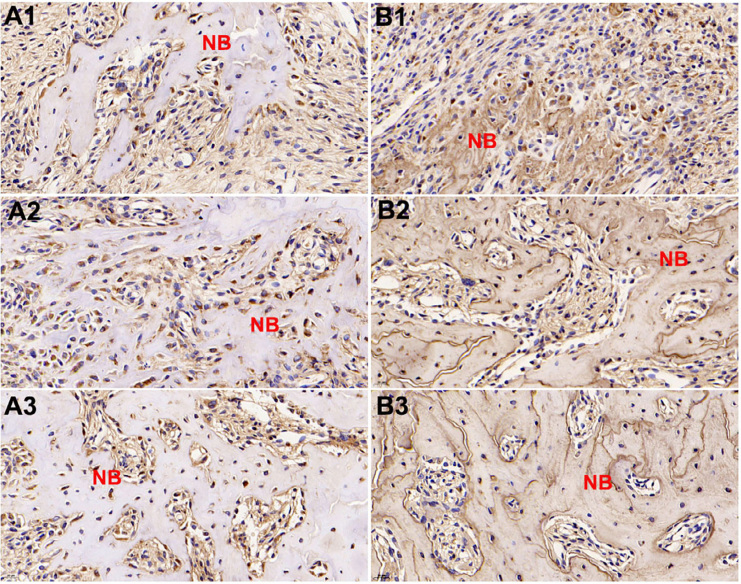
Representative immunostaining of OCN and OPN after tooth extraction. Increased expressions of OCN (A) and OPN (B) were observed during bone repair from two to eight weeks. Two weeks after operation (A1 and B1), four weeks after operation (A2 and B2), eight weeks after operation (A3 and B3). NB new bone (40 × magnification)

Increased expressions of OCN and OPN occurred with bone repair and mineralization. These proteins increased during bone repair from two to eight weeks after extraction, which agreed with micro-CT results. The positive expressions of OCN were detected in osteoblasts and osteocytes around and inside the newly trabecular bone ( Figure 4A ). For OPN, positive expressions were observed in newly formed trabecular bone matrix, osteoblasts and osteocytes ( Figure 4B ). (40 × magnification).

### Histological analysis of the alveolar socket after tooth extraction

Considering the micro-CT results, we included tooth extraction sockets with 1.5 weeks of healing in the histological analysis to observe the osteoclastic activity that caused resorption of the spire region of the alveolar ridge.

**Figure 5:**
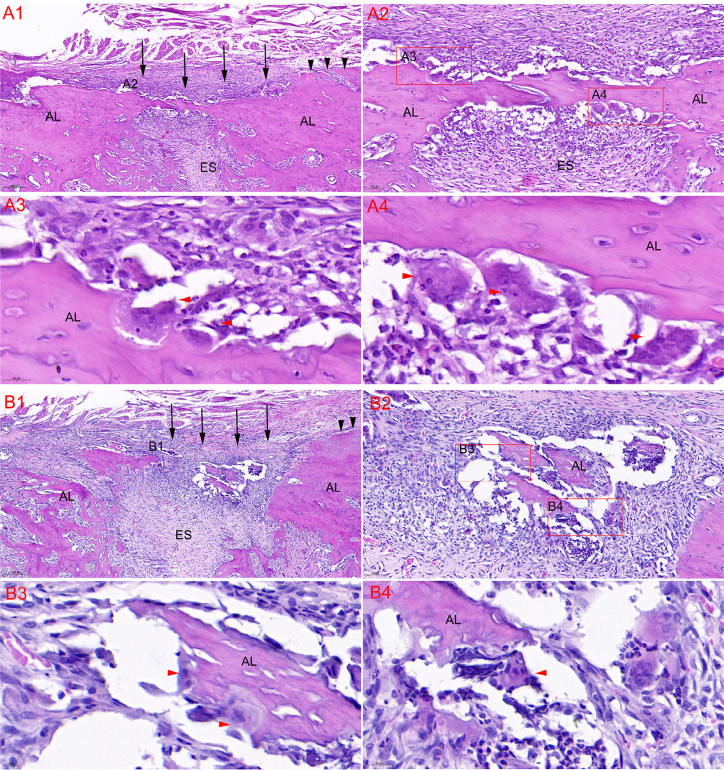
Histological images of the alveolar socket after extraction by HE staining. The height of lingual extraction socket bone wall (black arrows) was lower than that of the adjacent crestal region (black arrows) 1.5 weeks after extraction. Osteoclasts (red arrows) participated in the marked osteoclastic activity, which caused resorption of the crestal region of the alveolar (A1-A4). Inflammatory cells and multinucleated osteoclasts (red arrows) were found around the small crestal bone islands, which had not been completely absorbed 1.5 weeks after extraction (B1-B4). AL alveolar bone, ES extraction socket. A1 and B1 (8 × magnification), A2 and B2 (20 × magnification), A3 and B4 (72 × magnification), A4 and B3 (83 × magnification)

**Figure 6:**
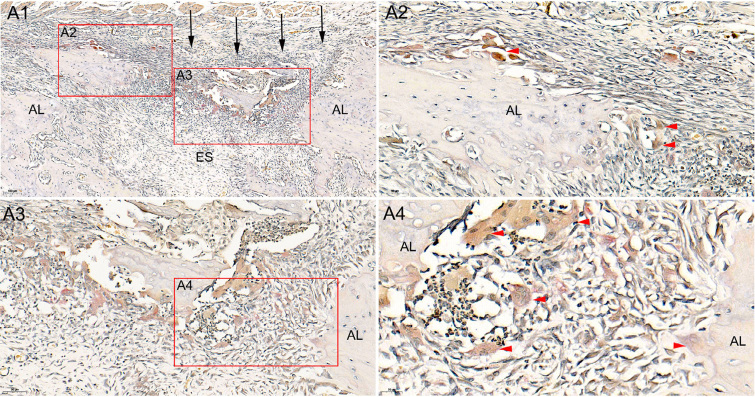
Histological images of the alveolar socket after extraction by TRAP staining. Trap-positive multinucleated osteoclasts were found around the small bone islands, which had not been completely absorbed 1.5 weeks after extraction. A1 (9 × magnification), A2 (27 × magnification), A3 (33 × magnification), A4 (53 × magnification)

Many multinucleated cells were found around the bone wall of the lingual extraction socket ( Figure 5 ). The height of lingual extraction socket bone wall was lower than that of the adjacent crestal region 1.5 weeks after extraction. The multinucleated cells were identified as osteoclasts by TRAP staining ( Figure 6 ).

**Figure 7:**
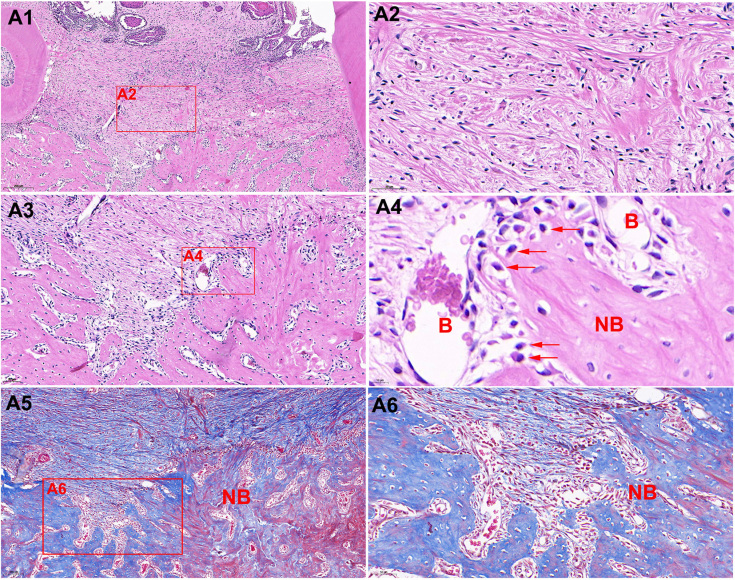
Histological images of the alveolar socket three weeks after extraction by HE staining. Few inflammatory cells and multinucleated osteoclasts were found (A1) (8×). The extraction socket was filled with many fibroblasts and collagen fiber bundles (A2) (36 × magnification). New trabecular bone (NB) and blood vessels (B) filling most of the alveolar socket were observed (A3) (30 × magnification). Osteoblasts (red arrows) can be seen at the edge of new bone tissues(A4) (86 × magnification). Masson’s staining revealed new bone form and mineralization. The red dye indicates mineralized bone and blue dye indicates mineralizing bone (A5 and A6). A5 (10 × magnification), A6 (27 × magnification)

We also included tooth extraction sockets with three weeks of healing to observe bone repair. Few inflammatory cells and multinucleated osteoclasts were found. The extraction socket was filled with many collagen fiber bundles and new bone trabeculae. Many blood vessels and osteoblasts were found at the edge of new bone tissue. Masson’s trichrome staining also revealed that a substantial amount of mineralized new bone was formed in the extraction socket ( Figure 7 ).

### Expression levels of EMMPRIN and its downstream MMP-9 in the alveolar ridge absorption

**Figure 8:**
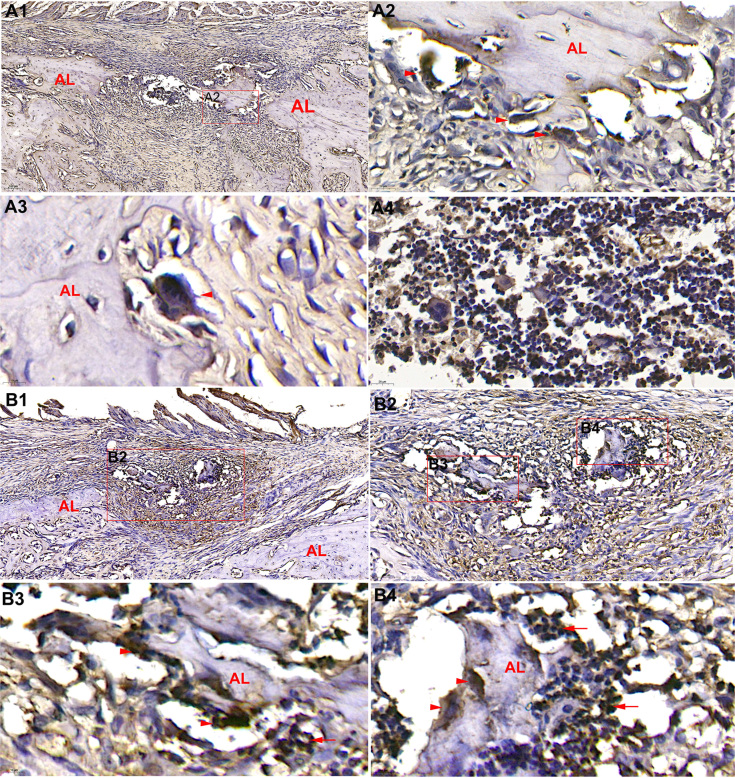
Representative immunostaining of EMMPRIN and MMP-9 after tooth extraction. EMMPRIN-positive osteoclasts participated in the marked osteoclastic activity, which caused resorption of the crestal region of the alveolar (A1) (10 × magnification). EMMPRIN-positive osteoclasts (red arrows) were observed in the lacuna resorption pits (A2 and A3) (63 × and 110 × magnification). Many positive-monocytes and multinucleated cells in bone marrow cavity were found 1.5 weeks after tooth extraction (A4) (110 × magnification). MMP-9-positive osteoclasts (red arrows) and inflammatory cells (red arrows) were observed during the resorption of the crestal region of the alveolar bone wall (B1-B4). B1 (10 × magnification), B2 (28 × magnification), B3 and B4 (110 × magnification)

EMMPRIN-positive osteoclasts resulted in the absorption of the spire region of the alveolar bone ( Figure 8 A1 - A4 ). The osteoclasts around the alveolar ridge were also MMP-9-positive. These results revealed that EMMPRIN and its downstream MMP-9 were involved in the reduced height of the extraction socket lingual bone wall ( Figure 8 B1 - B4 ).

## Discussion

Tooth extraction typically leads to loss of ridge height and width. Thus, it is essential to understand the following tissue alterations.^26^ The buccal-lingual loss of the ridge was reduced by at least 50% during the first year after human premolar or molar extraction, and 30% of the initial ridge width was lost during the first three months.^27^ Beagle dogs need four to six months of healing to show alveolar repair. Tooth extraction in rats with shorter healing time is cheaper and easier compared to studies in humans and in dogs. Eight weeks of healing is usually applied to observe alveolar repair in rats. To our knowledge, this study was the first to assess the absorption and healing processes of rat mandibular second molar extraction socket with significantly different buccal and lingual alveolar ridge width. The crestal region of the buccal bone wall was thicker than that of the lingual bone wall in mandibular second molars. Mandibular second molars could be fully extracted using a minimally invasive procedure.

We used the micro-CT to evaluate the bone microstructure in a non-destructive three-dimensional way. According to the micro-CT results, the outline of the extraction socket was evident immediately after extraction. New bone trabeculae filled the apical third of the alveolar socket two weeks after operation. The alveolar socket was largely filled with new bone trabeculae after four weeks. The extraction socket was fully filled with new bone after eight weeks. Bone morphological parameters, including BV/TV, BMD, and Tb.Th, which were positively correlated with bone load capacity and quality, progressively from two to eight weeks. The values decrease when osteoporosis, fracture and bone resorption occur. We observed the osteogenic marker OCN and OPN to evaluate bone healing from two to eight weeks. Consistent with micro-CT results, the expressions of OCN and OPN increased during bone repair after extraction. The healing process agreed with many previous reports.^5 , 12 , 15 , 16^

We included HE and Masson’s staining results of the extraction sockets with three weeks of healing to observe bone repair and to further certify the micro-CT results of four weeks of healing. The extraction sockets showed a substantial amount of mineralizing new bone and collagen fiber bundles. We found few inflammatory cells and multinucleated osteoclasts.

Bone alterations of the residual alveolar bone wall, including serious bone changes in the height and width, followed the tooth extraction. We paid more attention to the height alteration of the alveolar ridge after extraction because the lack of height of alveolar bone hinders the application of dental implants. Researchers must know how the alveolar crest changes when different teeth are removed. The lingual wall height of the extraction socket decreased significantly two weeks after extraction. However, the buccal wall height was rarely reduced after extraction. Bone absorption in the crestal region of the bone wall near the buccal side was mainly found near the extraction socket side. The width of the alveolar ridge was similar between the buccal and lingual sides in most tooth extraction models. However, rat mandibular second molar showed a significant width difference; the buccal wall was more than twice as thick as the lingual wall.

The lingual wall height did not increase significantly, although bone volume and trabecular thickness increased from two to eight weeks after extraction. The reduced height of bone wall near the lingual side of the extraction socket was rarely repaired in the later repair stage. Our results also showed that alveolar ridge width was a determinant of alveolar ridge loss after tooth extraction.^27 - 30^ The relationship between ridge width and height loss was another focus of the study.

We included tooth extraction sockets with 1.5 weeks of healing to observe osteoclastic activity that caused lingual alveolar ridge site loss. We found many TRAP-positive osteoclasts around the alveolar ridge site. Residual lingual alveolar ridge height was mainly determined by bone resorption and remodeling within two weeks. Thus, further studies are needed on reducing alveolar ridge site loss by inhibiting osteoclastic activity two weeks after extraction of mandibular second molars.

The levels of EMMPRIN and MMP-9 have been identified as markers of cancer and inflammatory diseases, being critical to regulate inflammation and bone metabolism.^31^

We examined the expression levels of EMMPRIN and its downstream MMP-9 to certify if they participated in alveolar ridge absorption after tooth extraction.^32^ Immunohistochemical staining showed that both EMMPRIN and MMP-9 were positively expressed in the osteoclasts that participated in resorption of the crestal region of the alveolar bone. More attention should be paid to reducing alveolar ridge site loss by inhibiting osteoclastic activity within two weeks after the mandibular second molars extraction. EMMPRIN and MMP-9 signaling may offer a potential therapeutic target to attenuate bone resorption. Molecular mechanisms of EMMPRIN and MMP-9 in bone resorption after tooth extraction require further research.

The goal of alveolar ridge site preservation is to preserve as much of the alveolar bone of the extraction wound as possible before implanting, ensuring that the implant has more bone to support it.^33 , 34^ Studies have mostly focused on increasing bone regeneration after tooth extraction.^35 - 38^ The repair of unilateral alveolar ridge site loss is easier than that of buccal-lingual bilateral loss. Only lingual bone wall height was reduced in the rat mandibular second molar extraction model. This study offers a new model for future study on alveolar ridge site preservation after tooth extraction.

## Conclusions

1) The rat mandibular second molar extraction model suits the assessment of alveolar ridge absorption and preservation. 2)The EMMPRIN–MMP-9 pathway may be a candidate for further study on attenuating bone resorption after tooth extraction.
